# Supplementary material for: SPaRTAN, a computational framework for linking cell-surface receptors to transcriptional regulators
Source: Nucleic Acids Res. 2021 Sep 9;49(17):9633–47. doi: 10.1093/nar/gkab745 (PMC8464045; doi:10.1093/nar/gkab745)
Supplement: gkab745_Supplemental_File [file gkab745_supplemental_file.pdf]

Supplementary material: a computational framework for linking cell-surface receptors to transcriptional regulators

Supplementary Figures

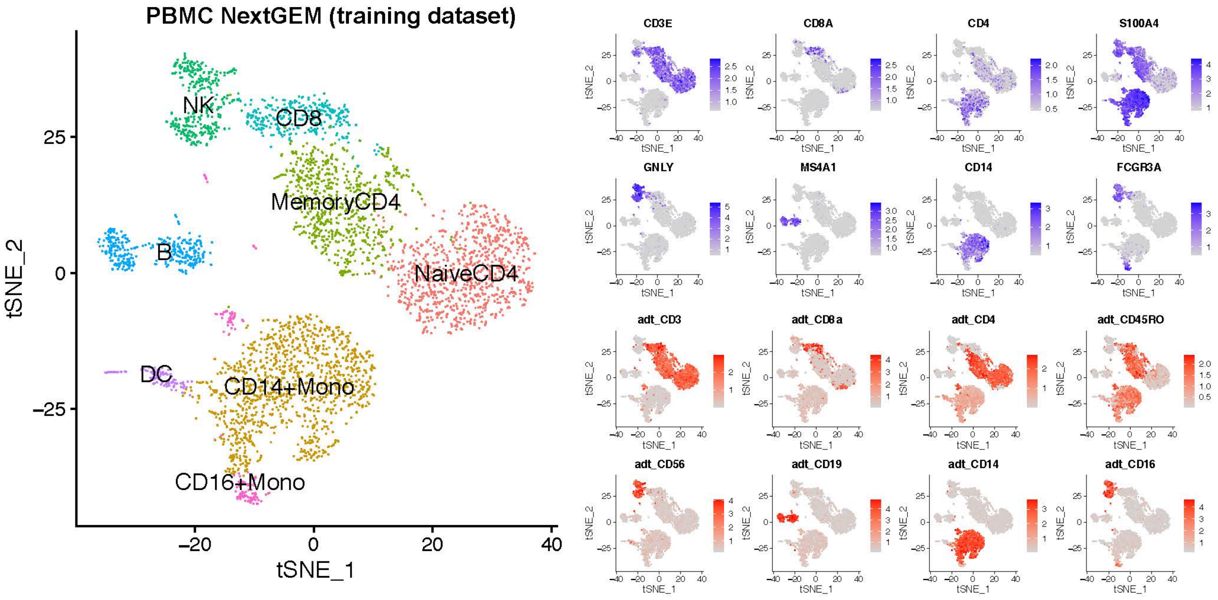

Supplementary Fig. 1: Transcriptome-based clustering of 4,073 CITE-seq single-cell expression profiles of PBMCs (Next GEM, training dataset) reveals distinct cell populations. Cell types can be discerned by marker gene expression. B, $CD20^{+}CD19^{+}$ B cells; Naïve CD4, $CD62L^{+}CD45RA^{+}CCR7^{+}CD4^{+}$ T cells; Memory CD4, $CD62L^{-}CD45RA^{-}CCR7^{-}CD4^{+}CD3^{+}$ T cells; CD8 T, $CD8^{+}CD3^{+}$ T cells; NK, $CD56^{+}CD3^{+}CD4^{-}CD8^{-}$cells; CD14+ Mono, $CD14^{+}$HLA-$DR^{+}CD3^{-}CD19^{-}$; CD16+ Mono, $CD16^{+}$HLA-$DR^{+}CD3^{-}CD19^{-}$; DC, $CD14^{-}HLADR^{+}CD123^{-}CD11c^{+}$. mRNA (blue) and corresponding ADT (red) signal for the CITE-seq antibody panel projected on the t-SNE plot from the panel.

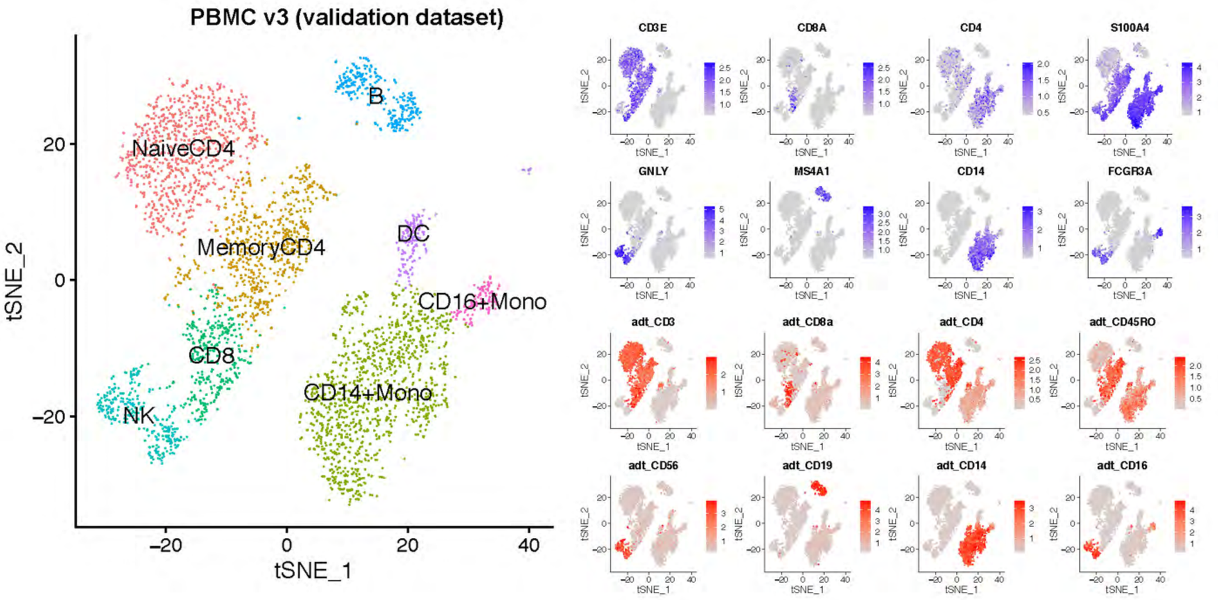

Supplementary Fig. 2: Transcriptome-based clustering of 3,891 CITE-seq single-cell expression profiles of PBMCs (10x Genomics, v3 Chemistry, validation dataset) reveals distinct cell populations. Cell types can be discerned by marker gene expression. B, $CD20^{+}CD19^{+}$ B cells; Naïve CD4, $CD62L^{+}CD45RA^{+}CCR7^{+}CD4^{+}$ T cells; Memory CD4, $CD62L^{-}CD45RA^{-}CCR7^{-}CD4^{+}CD3^{+}$ T cells; CD8 T, $CD8^{+}CD3^{+}$ T cells; NK, $CD56^{+}CD3^{+}CD4^{-}CD8^{-}$cells; CD14+ Mono, $CD14^{+}$HLA-$DR^{+}CD3^{-}CD19^{-}$; CD16+ Mono, $CD16^{+}$HLA-$DR^{+}CD3^{-}CD19^{-}$; DC, $CD14^{-}HLADR^{+}CD123^{-}CD11c^{+}$. mRNA (blue) and corresponding ADT (red) signal for the CITE-seq antibody panel projected on the t-SNE plot from the panel.

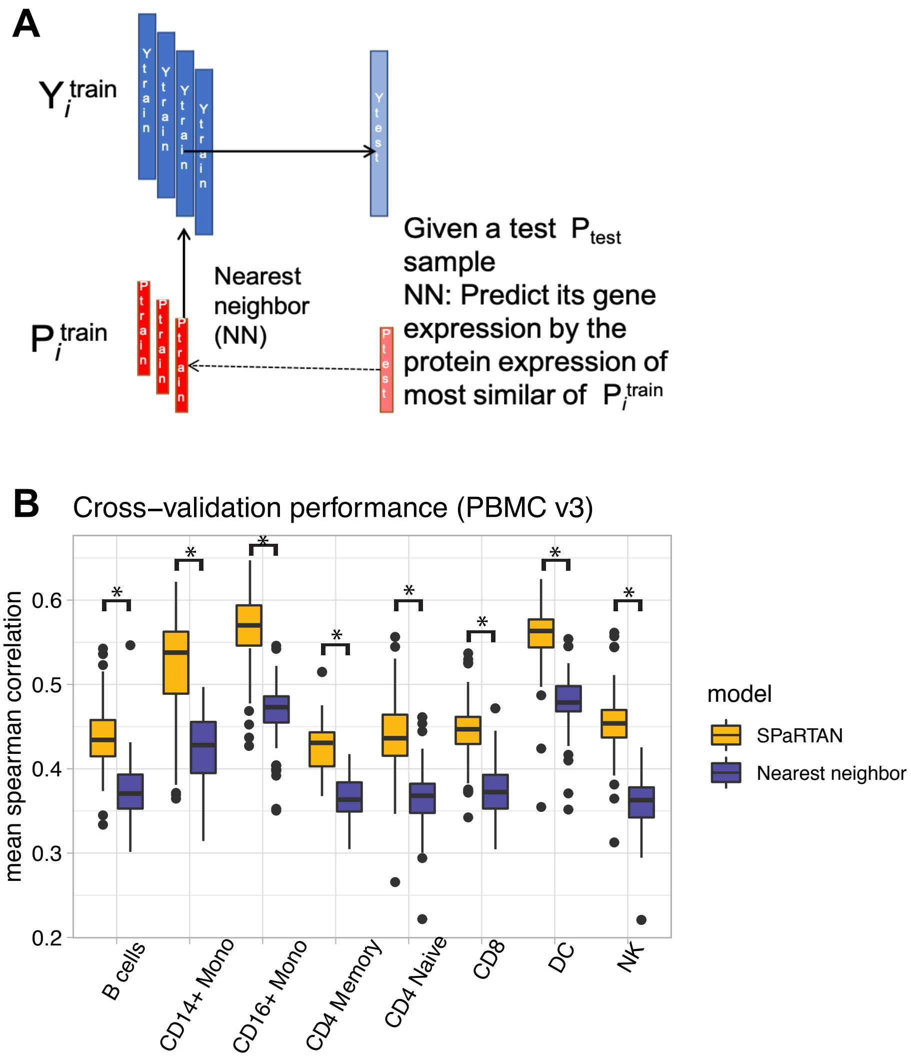

Supplementary Fig. 3: (A) Representative figure for nearest neighbor approach. Neighbors are chosen based on similarity of protein expression profiles (input space). In fivefold cross-validation on held-out cells, based on protein expression nearest neighbor, where the training domain that is most similar to each test example on the basis of protein expression is considered the nearest neighbor, and this neighbor's gene expression is used for prediction. We used Euclidean distance in the protein space to identify the nearest neighbor. Essentially, high accuracy for the prediction problem is not the critical goal but rather being able to learn important molecular connections in diverse cellular contexts, specifically the connectivity of surface receptors and TFs whose activity explains gene expression differences among cells. Therefore, we mainly want to show that the model does more than memorize obvious cell-to-cell similarity in the training data (hence we use the nearest neighbor comparison). **(B)** SPaRTAN accurately predicts relative gene expression on held-out PBMC (10x Genomics, v3 Chemistry, validation dataset) cells for each cell-type. Performance of the SPaRTAN models for each PBMC cell type compared to nearest neighbor methods. Boxplots showing mean Spearman correlations between predicted and actual gene expression using the SPaRTAN model (light blue); nearest neighbor by surface protein expression profile (blue) (*y*-axis) for PBMC CITE-seq data from 10x Genomics (v3 Chemistry) each cell-type (P<0.001, one-sided Wilcoxon's signed-rank test).

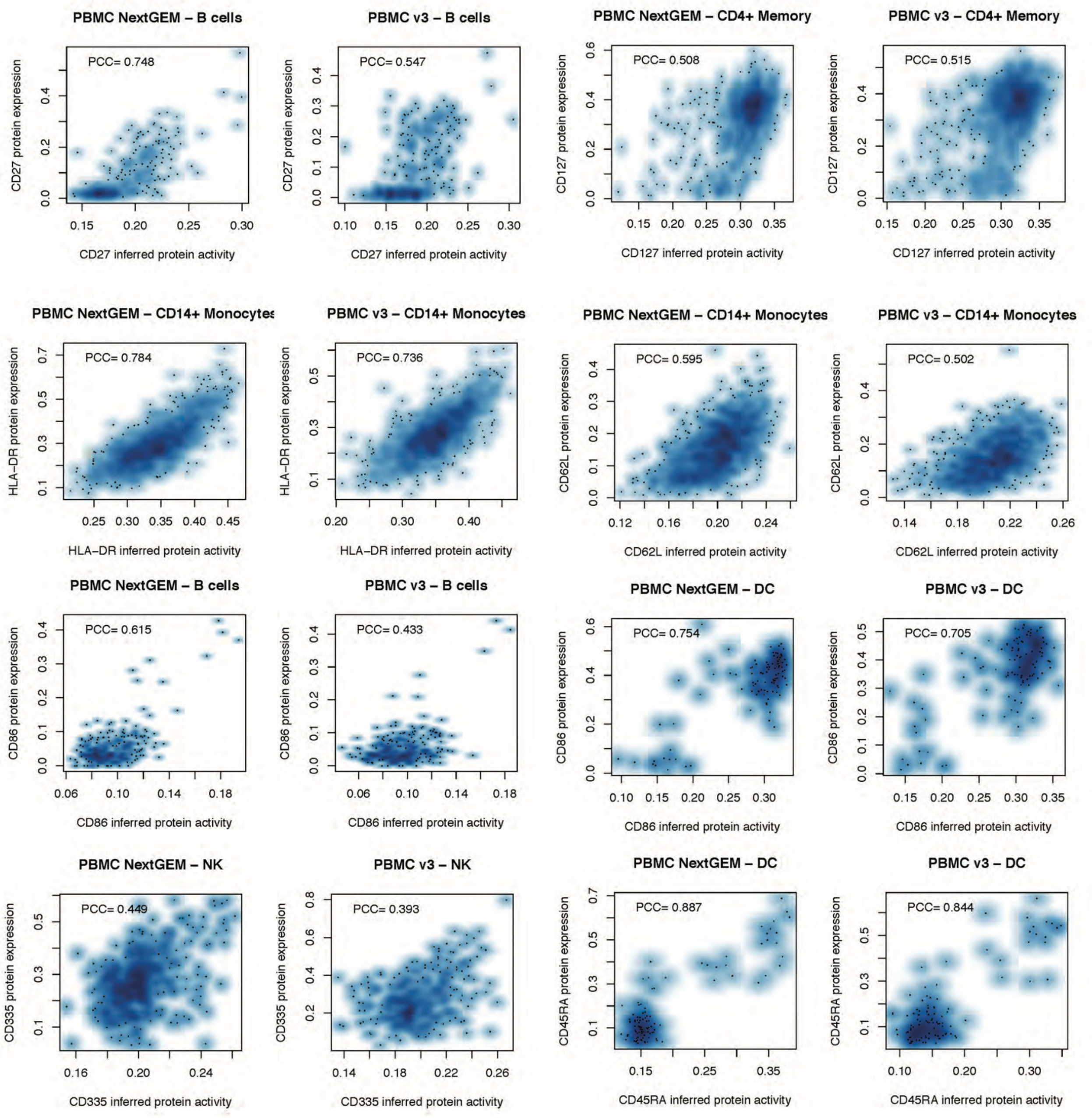

Supplementary Fig. 4: Correlation of inferred and measured protein expression. Pearson's correlation coefficient (PCC) of inferred and measured surface protein expression in training PBMC cohort (left) and validation PBMC cohort (right); for validation data, we predict protein expression using the SPaRTAN cell type model on PBMC (NextGEM) and measure protein expression using the PBMC (V3 Chemistry) resource.

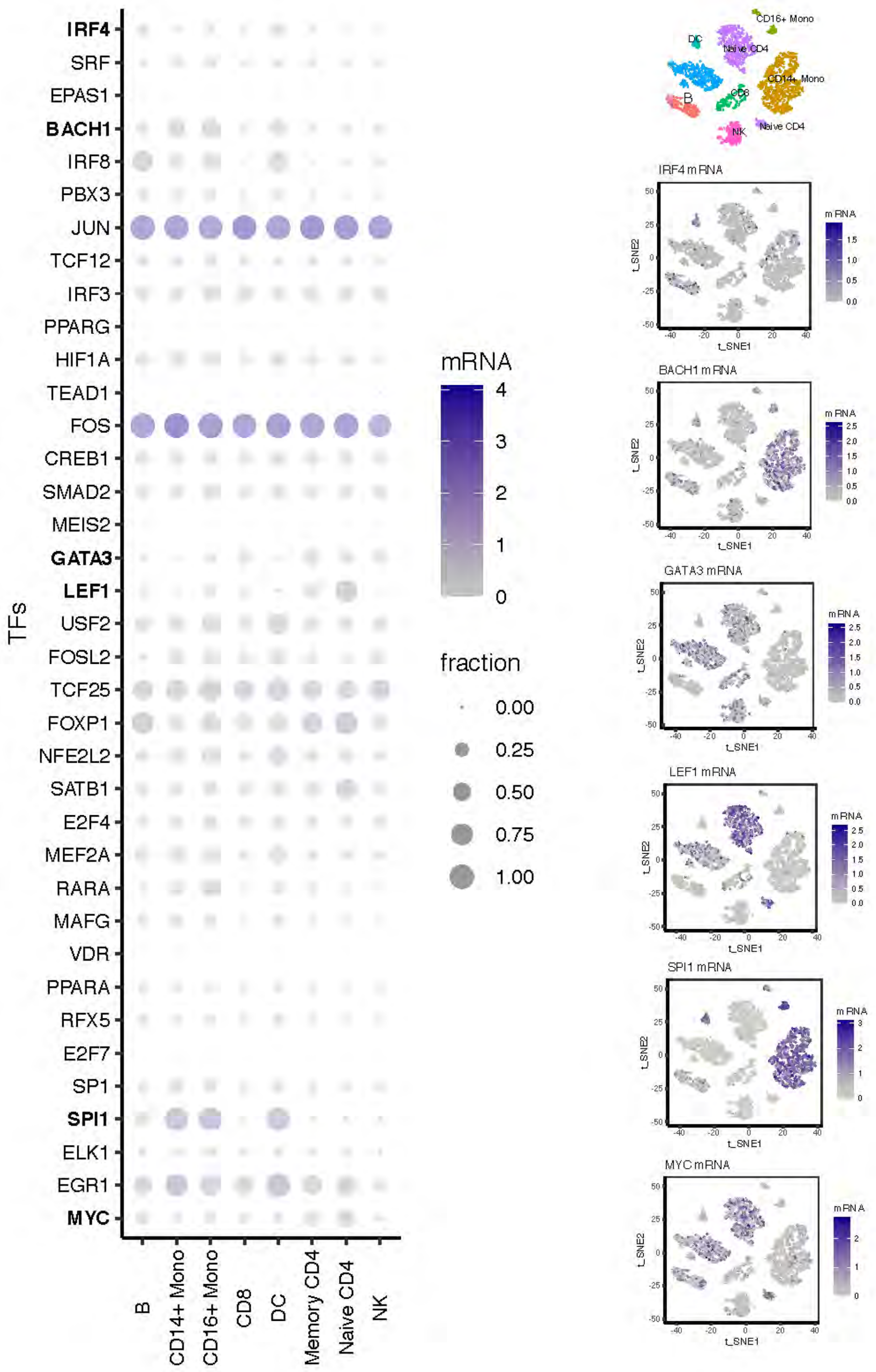

Supplementary Fig. 5: Dot plot showing the median TF mRNA expression z-score of TFs across different PBMC cell-types. The dot size indicates a fraction of cells each TF is identified as a significant regulator within each cell-type from inferred TF activities (**Fig 1E**). t-SNE on the inferred TF activity matrix. Cells are colored according to major cell types. IRF4, BACH1, GATA3, LEF1, SPI1 and MYC mRNA expression overlay on t-SNE of TF activities.

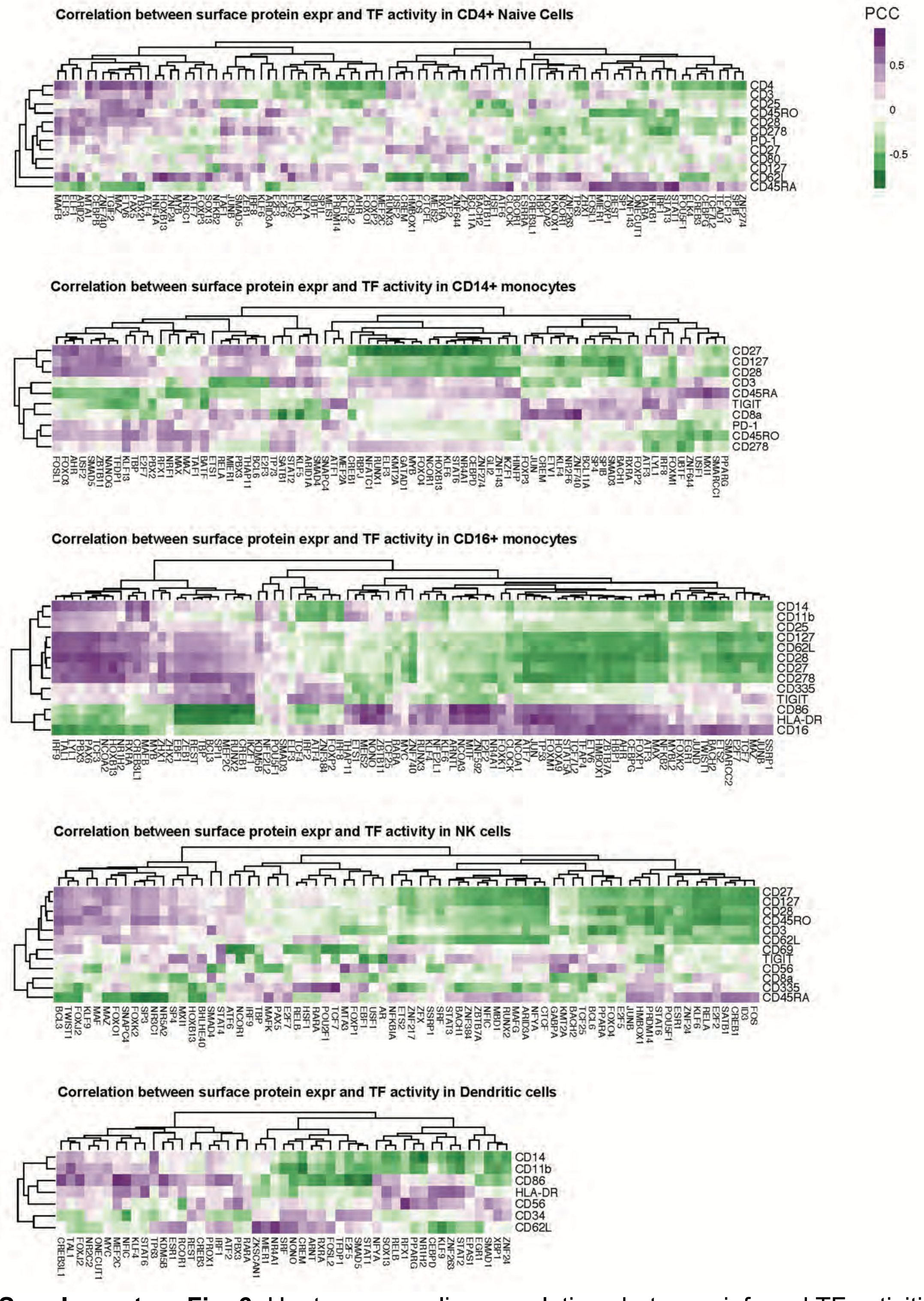

Supplementary Fig. 6: Heatmap revealing correlations between inferred TF activities of cells (columns) and surface protein expression (rows) in CD4^{+} naïve T cells, CD14^{+} monocytes, CD16^{+} monocytes, NK and Dendritic cells. For clarity, surface proteins with pairwise Pearson correlation values with TFs below 0.75 are filtered, and then the union of the top 10 most correlated TFs with each surface is shown for each cell type.

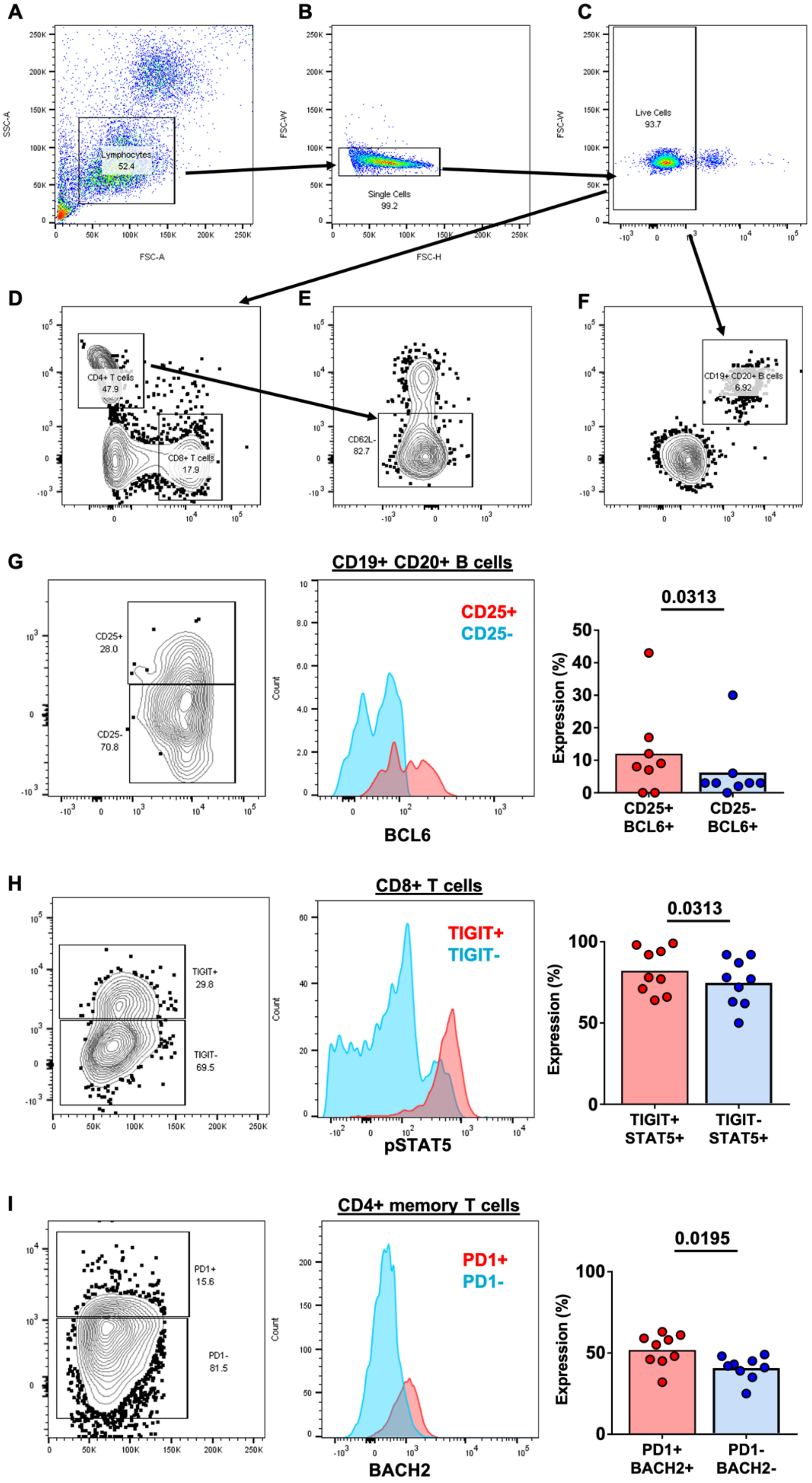

A
B
C
D
E
F
G
H
I
Lymphocytes
52.4
Single Cells
99.2
Live Cells
93.7
CD4+ T cells
47.9
CD8+ T cells
17.9
CD62L-
82.7
CD19+ CD20+ B cells
6.92
CD19+ CD20+ B cells
CD25+
28.0
CD25-
70.8
CD25+
CD25-
BCL6
0.0313
Expression (%)
CD25+ BCL6+
CD25- BCL6+
CD8+ T cells
TIGIT+
29.8
TIGIT-
69.5
TIGIT+
TIGIT-
pSTAT5
0.0313
TIGIT+ STAT5+
TIGIT- STAT5+
CD4+ memory T cells
PD1+
15.6
PD1-
81.5
PD1+
PD1-
BACH2
0.0195
PD1+ BACH2+
PD1- BACH2-

Supplementary Fig. 7: Flow cytometric analysis and validation of inferred TF activity and surface protein expression associations in healthy donor PBMCs. **(A)** Gating strategy representations of lymphocytes, **(B)** singlets, **(C)** live cells, **(D)** $CD4^+$ T cells and $CD8^+$ T cells followed by **(E)** memory definition of $CD62L^-$ $CD45RA^-$ $CD4^+$ T cells, and **(F)** $CD19^+CD20^+$ B cells. **(G)** Representative flow gating of CD25 in B cells, along with representative histograms of BCL6 in the populations and distribution across samples (n=8). **(H)** Representative flow gating of TIGIT in $CD8^+$ T cells, along with representative histograms of pSTAT5 in the populations and distribution across samples (n=9). **(I)** Representative flow gating of PD-1 in $CD4^+$ memory T cells, along with representative histograms of BACH2 in populations and distribution across samples (n=9).

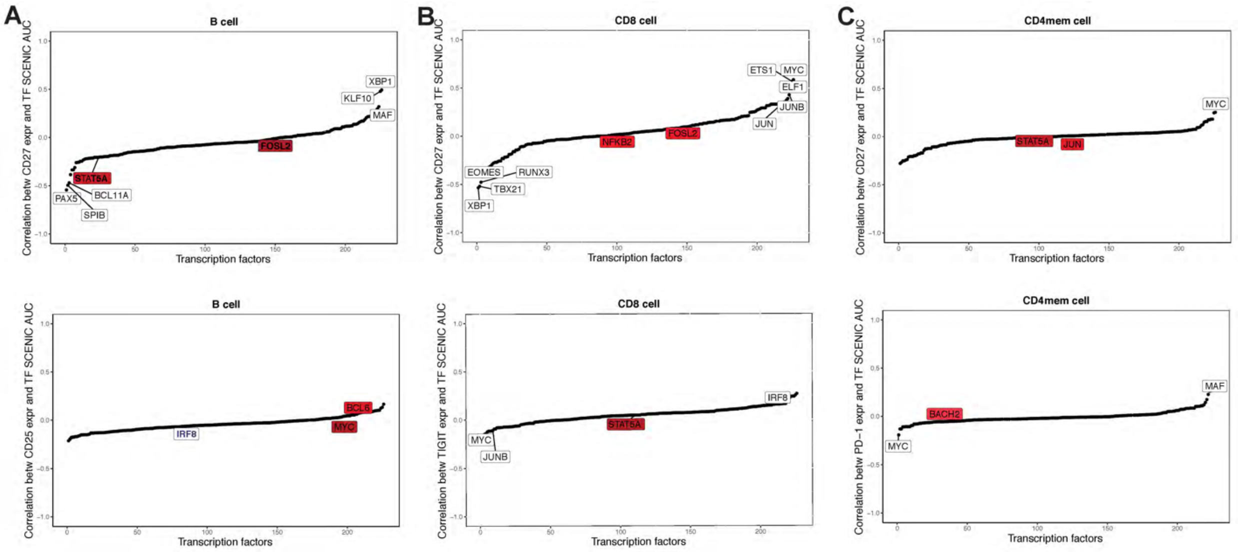

Supplementary Fig. 8: Correlation between **(A)** CD27 (top) CD25 (bottom) protein expression and SCENIC regulon activities across B cells; **(B)** CD27 (top) TIGIT (bottom) protein expression and SCENIC regulon activities across $CD8^+$ T cells; **(C)** CD27 (top) PD-1 (bottom) protein expression and SCENIC regulon activities across $CD4^+$ memory T cells.

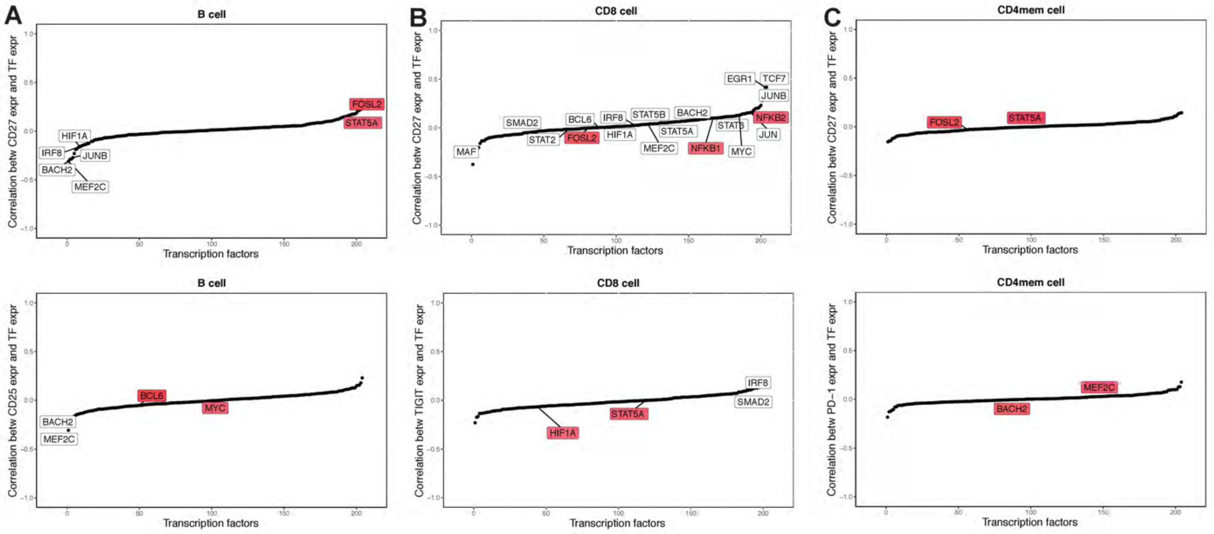

Supplementary Fig. 9: Correlation between **(A)** CD27 (top) CD25 (bottom) protein expression and TF mRNA expression across B cells; **(B)** CD27 (top) TIGIT (bottom) protein expression and TF mRNA expression across CD8^{+} T cells; **(C)** CD27 (top) PD-1 (bottom) protein expression and mRNA expression across CD4^{+} memory T cells.

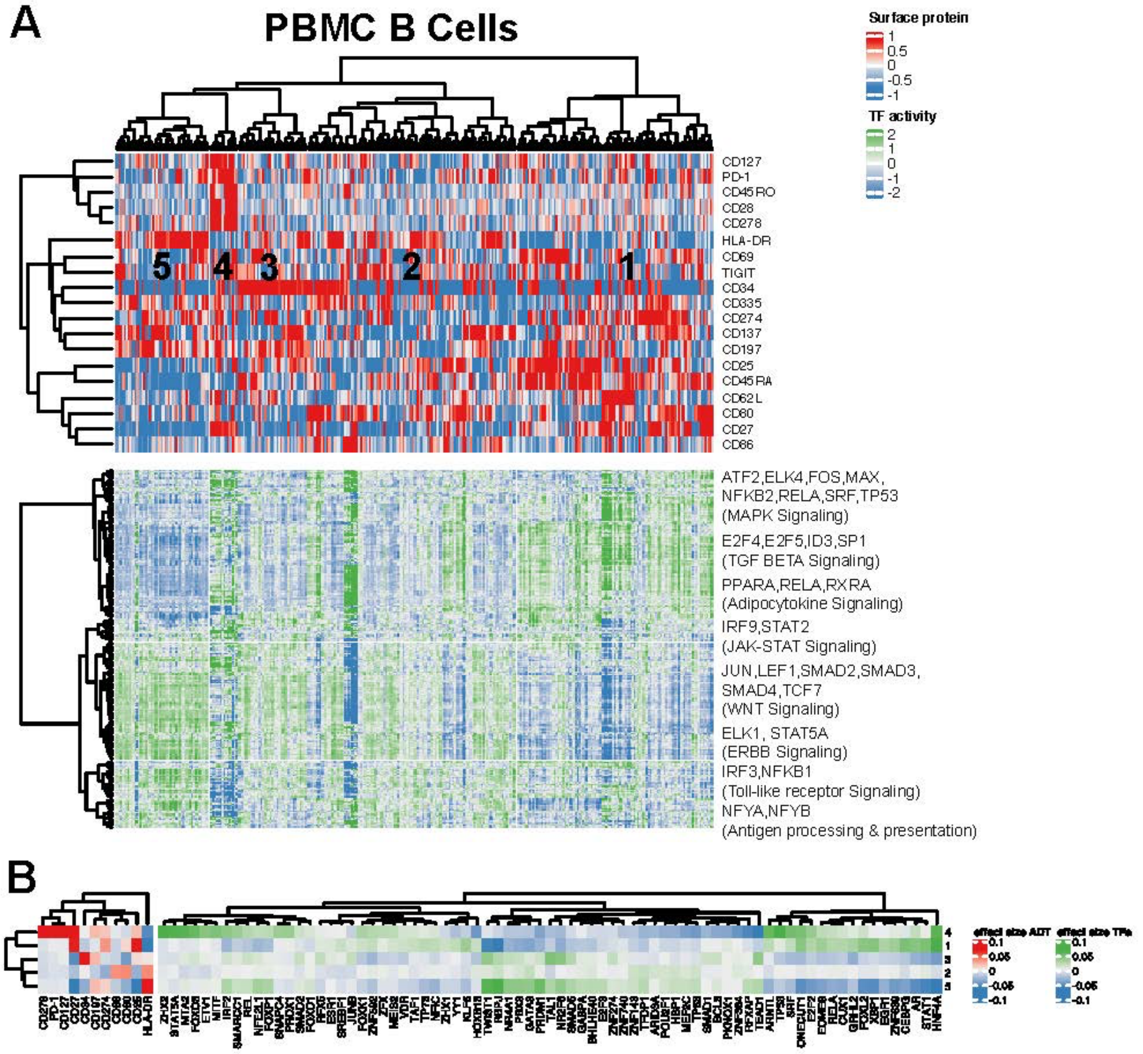

Supplementary Fig. 10: (A) We trained a SPaRTAN model on 332 B cells from the 10x Genomics PBMC CITE-seq dataset. The top heatmap shows cells clustered by the surface protein expression (excluding cell lineage maker surface proteins). The bottom panel shows inferred TF activities for each cell based on clustering by surface protein expression. **(B)** Heatmap shows the mean surface protein expression and inferred TF activity between cells in a given cluster vs. those in all other clusters. For each comparison, the absolute value of the mean surface protein expression and inferred TF activity (effect sizes) are ranked and the union of the top 10 TFs for each comparison is shown in the heatmap.

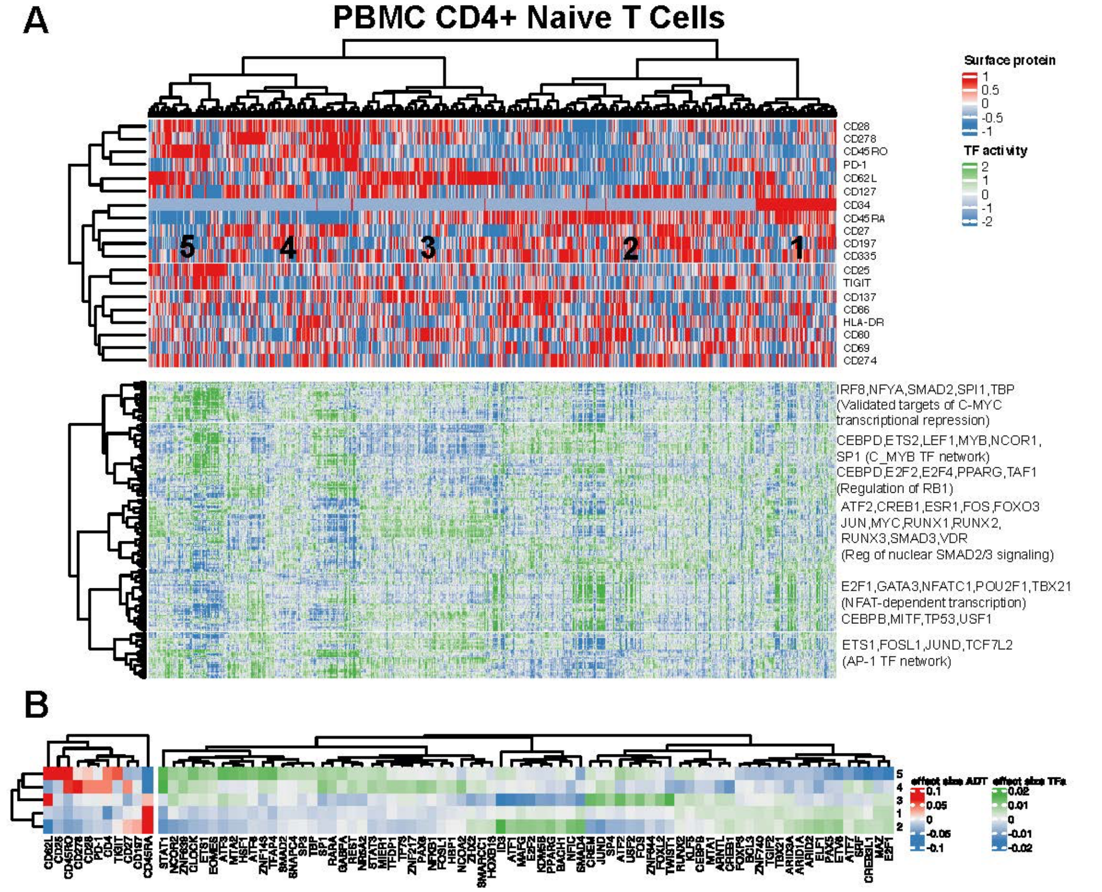

Supplementary Fig. 11: (A) We trained a SPaRTAN model on 910 CD4^{+} naïve T cells from the 10x Genomics PBMC CITE-seq dataset. The top heatmap shows cells clustered by the surface protein expression (excluding cell lineage maker surface proteins) The bottom panel shows inferred TF activities for each cell based on clustering by surface protein expression. **(B)** Heatmap shows the mean surface protein expression and inferred TF activity between cells in a given cluster vs. those in all other clusters. For each comparison, the absolute value of the mean surface protein expression and inferred TF activity (effect sizes) are ranked and the union of the top 10 TFs for each comparison is shown in the heatmap.

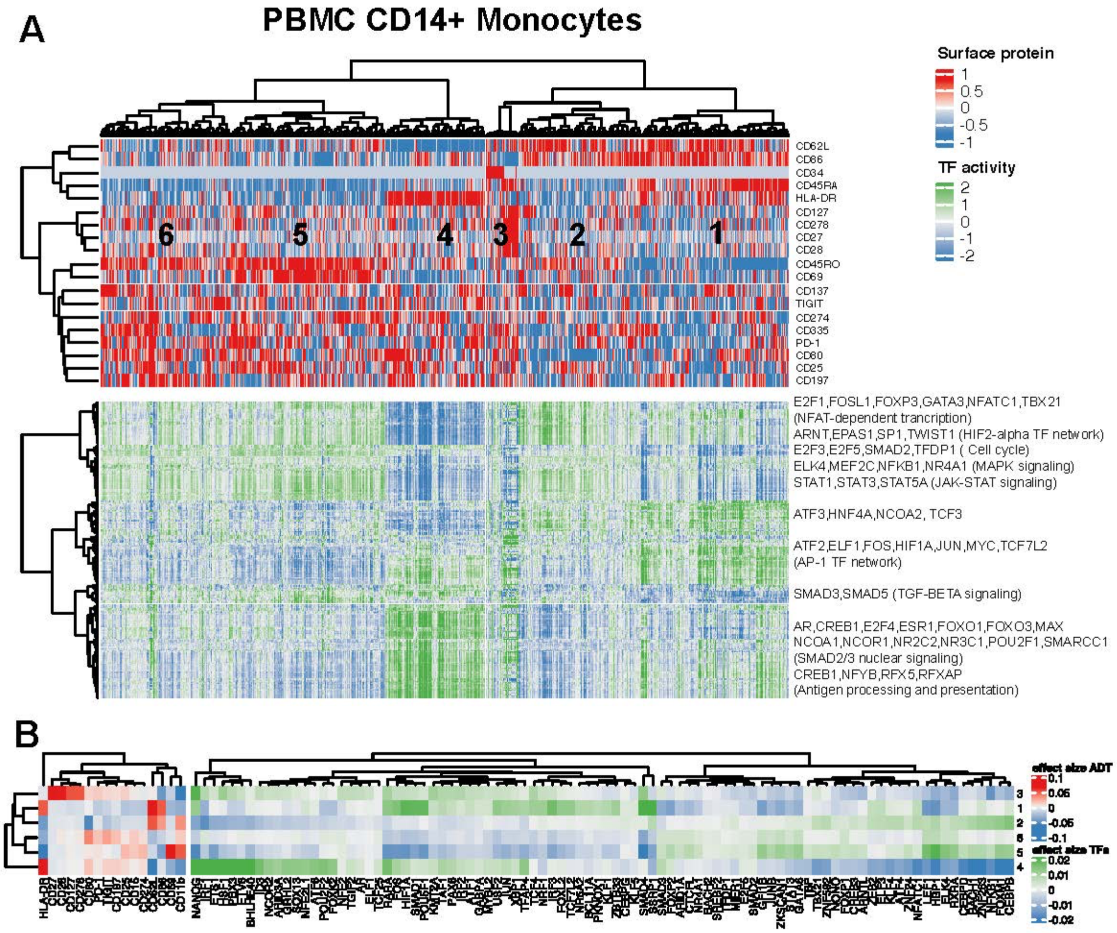

Supplementary Fig. 12: (A) We trained a SPaRTAN model on 1203 CD14[+] monocytes from the 10x Genomics PBMC CITE-seq dataset. The top heat map shows cells clustered by the surface protein expression (excluding cell lineage maker surface proteins) The bottom panel shows inferred TF activities for each cell based on clustering by surface protein expression. **(B)** Heatmap shows the mean surface protein expression and inferred TF activity between cells in a given cluster vs. those in all other clusters. For each comparison, the absolute value of the mean surface protein expression and inferred TF activity (effect sizes) are ranked and the union of the top 10 TFs for each comparison is shown in the heatmap.

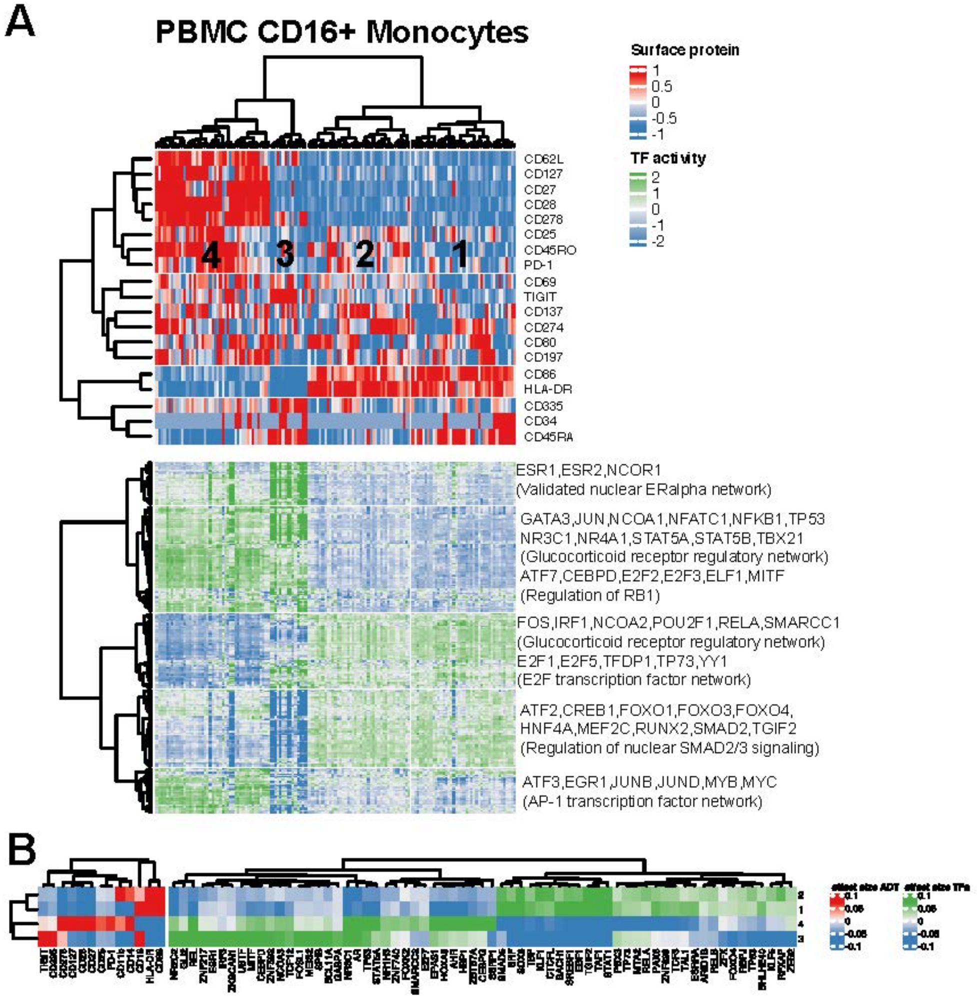

Supplementary Fig. 13: (A) We trained a SPaRTAN model on 140 CD16^{+} monocytes cells from the 10x Genomics PBMC CITE-seq dataset. The top heat map shows cells clustered by the surface protein expression (excluding cell lineage maker surface proteins) The bottom panel shows inferred TF activities for each cell based on clustering by surface protein expression. **(B)** Heatmap shows the mean surface protein expression and inferred TF activity between cells in a given cluster vs. those in all other clusters. For each comparison, the absolute value of the mean surface protein expression and inferred TF activity (effect sizes) are ranked and the union of the top 10 TFs for each comparison is shown in the heatmap.

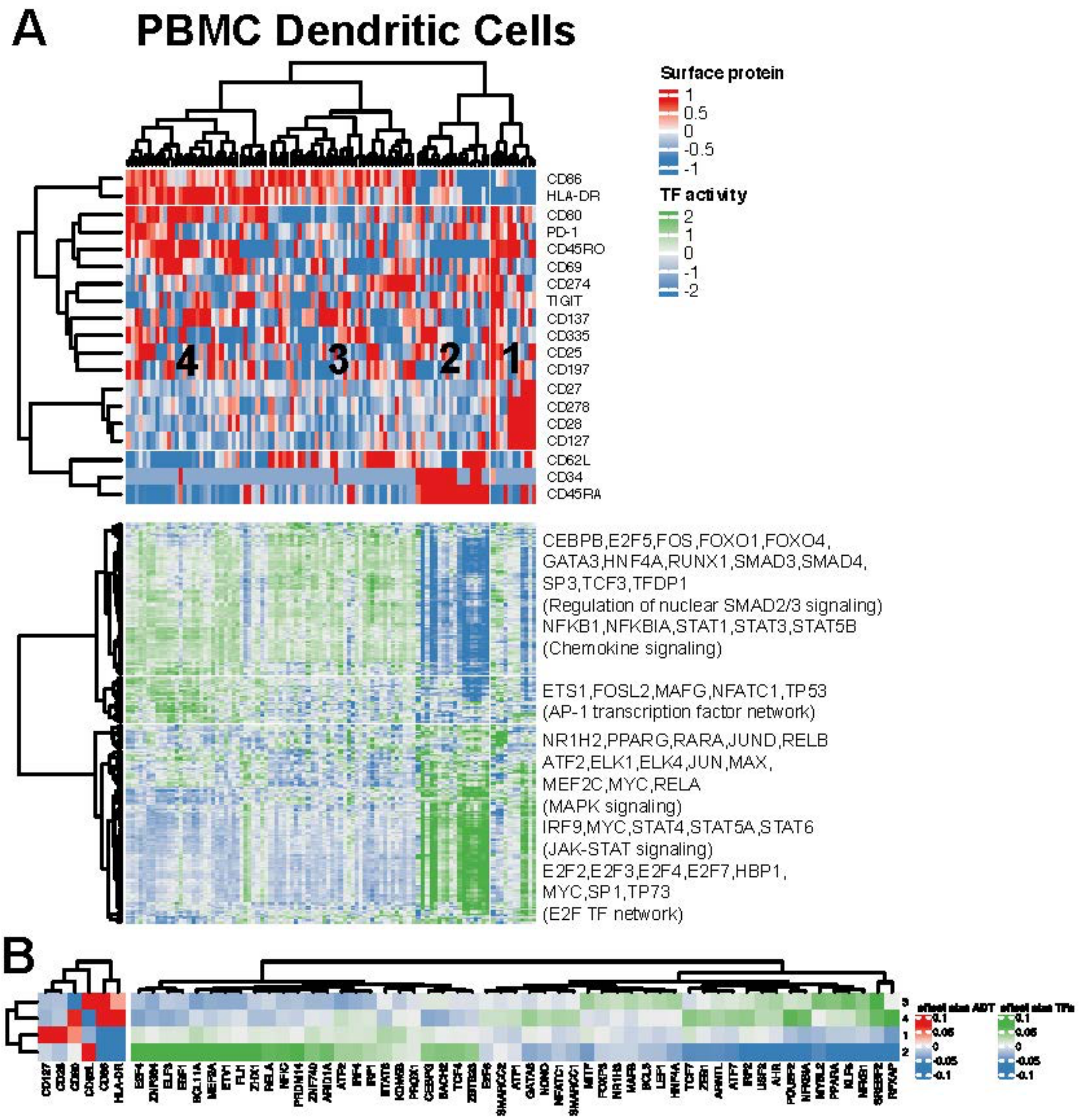

Supplementary Fig. 14: (A) We trained a SPaRTAN model on 100 dendritic cells from the 10x Genomics PBMC CITE-seq dataset. The top heatmap shows cells clustered by the surface protein expression (excluding cell lineage maker surface proteins) The bottom panel shows inferred TF activities for each cell based on clustering by surface protein expression. **(B)** Heatmap shows the mean surface protein expression and inferred TF activity between cells in a given cluster vs. those in all other clusters. For each comparison, the absolute value of the mean surface protein expression and inferred TF activity (effect sizes) are ranked and the union of the top 10 TFs for each comparison is shown in the heatmap.

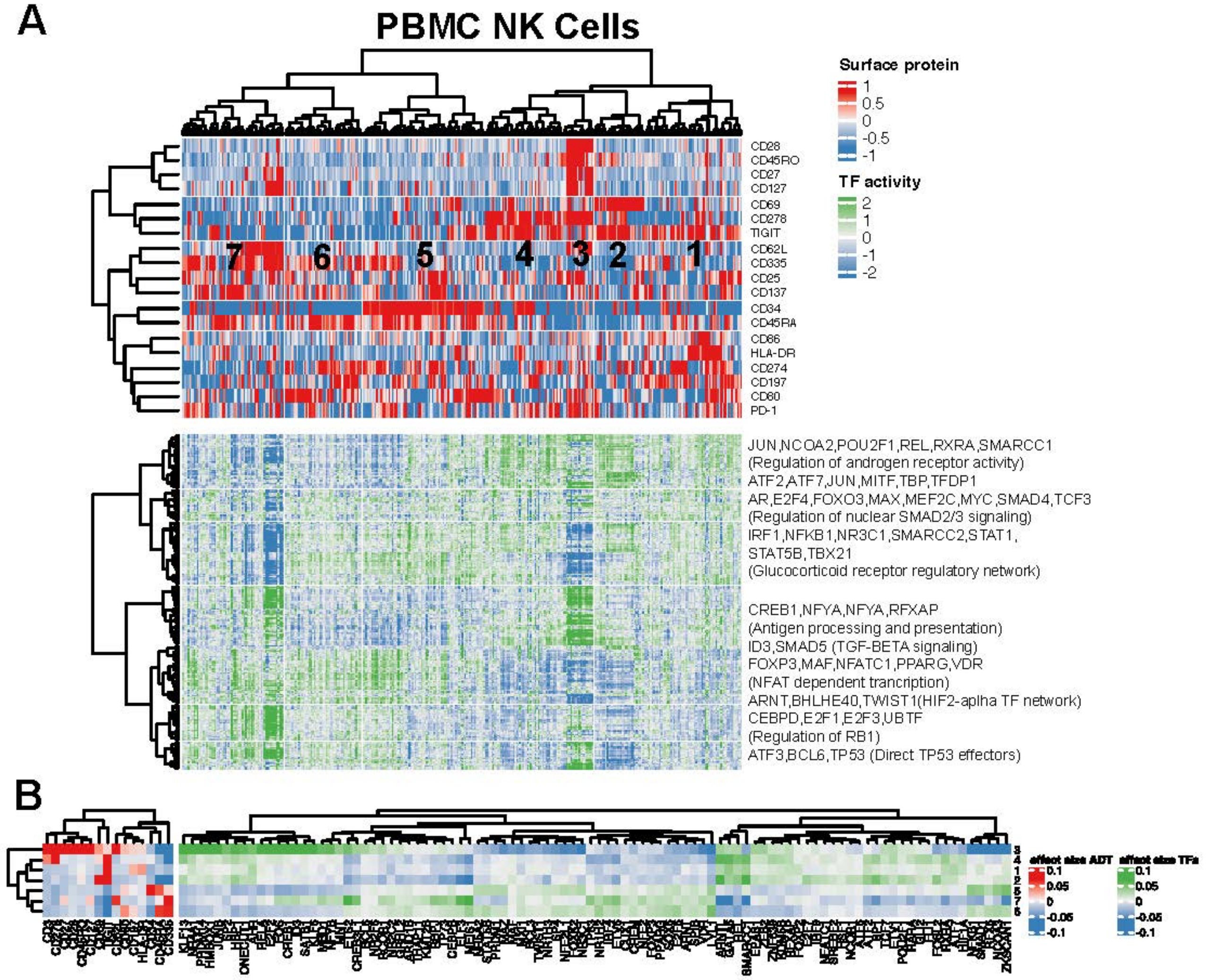

Supplementary Fig. 15: (A) We trained a SPaRTAN model on 353 NK cells from the 10x Genomics PBMC CITE-seq dataset. The top heatmap shows cells clustered by the surface protein expression (excluding cell lineage maker surface proteins) The bottom panel shows inferred TF activities for each cell based on clustering by surface protein expression. **(B)** Heatmap shows the mean surface protein expression and inferred TF activity between cells in a given cluster vs. those in all other clusters. For each comparison, the absolute value of the mean surface protein expression and inferred TF activity (effect sizes) are ranked and the union of the top 10 TFs for each comparison is shown in the heatmap.

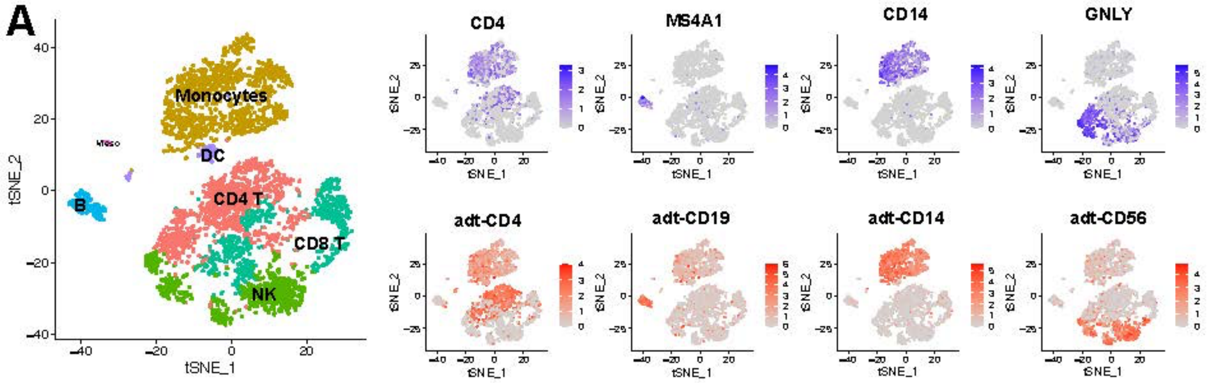

Supplementary Fig. 16: Transcriptome-based clustering of 4,005 CITE-seq single-cell expression profiles of malignant peritoneal mesothelioma (MPeM) reveals distinct cell populations. Cell types can be discerned by marker gene expression. B, B cells; CD4 T, $CD4^+$ T cells; CD8 T, $CD8^+$ T cells; NK, natural killer cells; Monocytes; DC, dendritic cells. mRNA (blue) and corresponding ADT (red) signal for the CITE-seq antibody panel projected on the t-SNE plot from the panel.

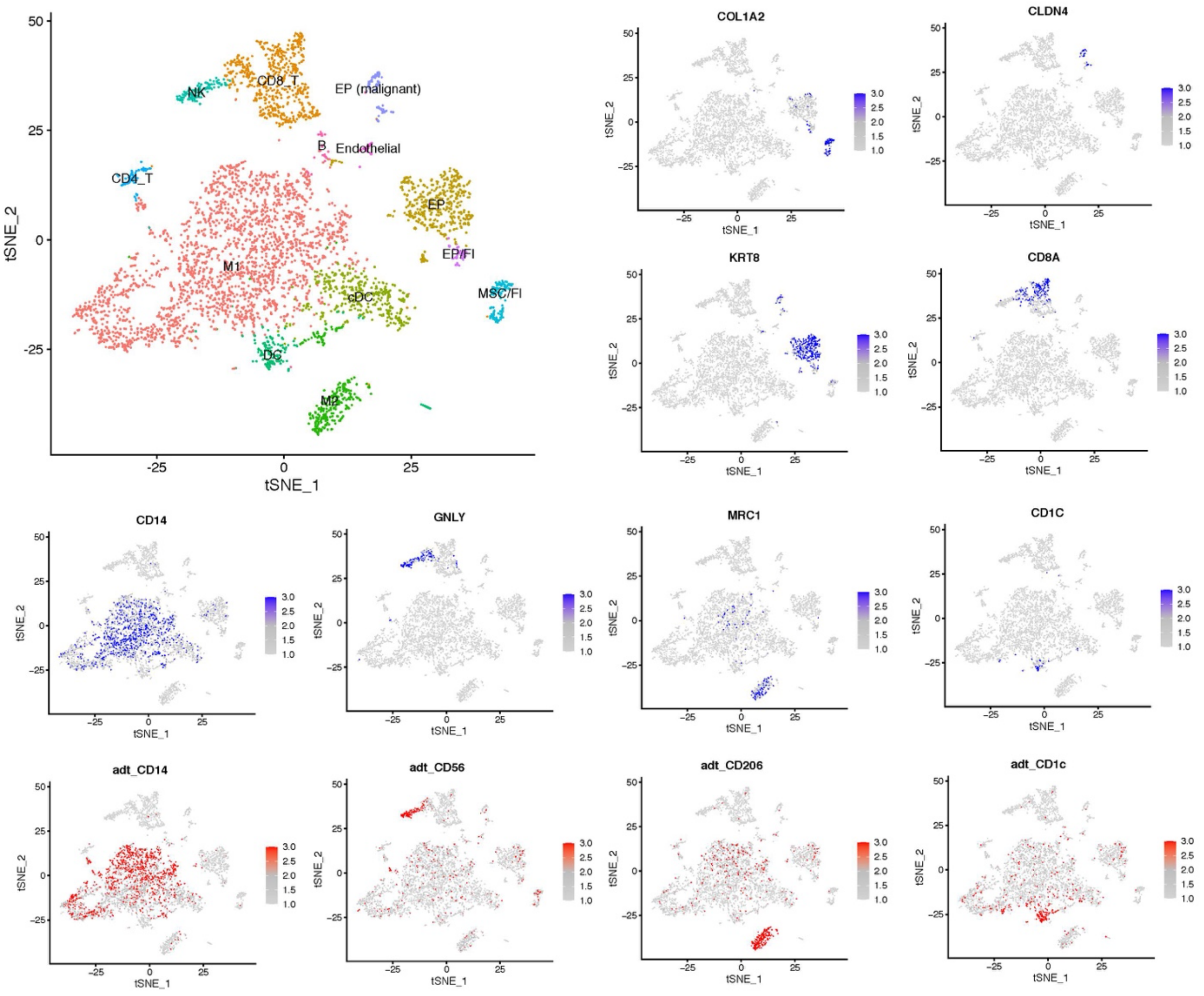

Supplementary Fig. 17: Transcriptome-based clustering of 4,912 CITE-seq single-cell expression profiles of malignant pleural mesothelioma (MPM) reveals distinct cell populations. Cell types can be discerned by marker gene expression. B, B cells; CD4 T, $CD4^{+}$ T cells; CD8 T, $CD8^{+}$ T cells; NK, natural killer cells; M1, M1 Macrophages; M2, M2 Macrophages; DC, dendritic cells; cDC, conventional DC; EP (malignant), epithelial malignant cells; Endothelial; EP, epithelial; EP/FI, epithelial and fibroblast; MSC/FI, mesenchymal stem cell and fibroblast. mRNA (blue) and corresponding ADT (red) signal for the CITE-seq antibody panel projected on the t-SNE plot from the panel.

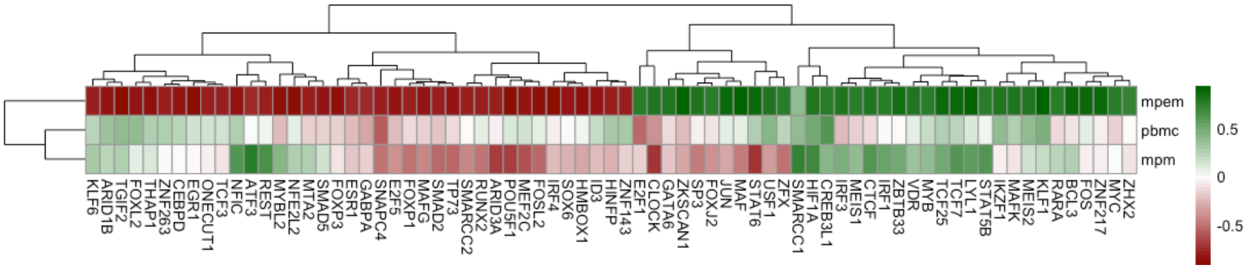

Supplementary Fig. 18: Heatmap revealing correlations between inferred TF activities of cells (columns) and PD-1 protein expression in PBMC, MPM and MPeM CD8$^+$ T cells. For clarity, TFs with pairwise Pearson correlation values with PD-1 below 0.75 in at least one tissue type are filtered.

Supplementary Tables

Supplementary Table 1. Summary of datasets used in this study

**Dataset**	**Summary**	**Reference**
CITE-seq (validation dataset)	*5k Peripheral blood mononuclear cells (PBMCs) from a healthy donor with cell surface proteins (v3 chemistry)*	https://support.10xgenomics.com/single-cell-gene-expression/datasets/3.1.0/5k_pbmc_protein_v3
CITE-seq (training dataset)	*5k Peripheral blood mononuclear cells (PBMCs) from a healthy donor with cell surface proteins (Next GEM)*	https://support.10xgenomics.com/single-cell-gene-expression/datasets/3.1.0/5k_pbmc_protein_v3_nextgem
CITE-seq (tumor dataset)	Malignant peritoneal mesothelioma	GSE accession number pending
CITE-seq (tumor dataset)	Malignant pleural mesothelioma	GSE accession number pending
DoRothEA database	Curated TF target-gene interaction	Garcia-Alonso et al.(1)

Supplementary Table 2. Pathway enrichment analysis for correlated TF-surface protein pairs across cell types using SPaRTAN and SCENIC frameworks

**Cell type**	**P-value - SPaRTAN**	**P-value - SCENIC**
B cells	**3.0e-06**	0.205
$CD14^{+}$ Monocytes	**2.38e-19**	0.185
$CD16^{+}$ Monocytes	**0.0126**	**0.0037**
$CD4^{+}$ Memory T cells	0.679	0.76
$CD4^{+}$ Naïve T cells	**0.014**	0.51
$CD8^{+}$ T cells	0.524	0.24
DC	**9.59e-25**	**1.92e-08**
NK	0.996	0.124

Supplementary Table 3. List of ab markers and clone for flow cytometry validation

Antigen	Clone	Catalog number	Company
CD8-BUV395	RPA-TA	563795	BD Biosciences
CD4-BUV737	SK3	612748	BD Biosciences
TIGIT- BV421	A15153G	372709	Biolegend
CD2-BV650	LG.3A10	124233	Biolegend
CD28-BV711	BC96	302635	Biolegend
CD45RA-BV785	HI100	304139	Biolegend
CD25-BV711	2A3	563159	BD Biosciences
CD20-BUV395	2H7	563781	BD Biosciences
CD19-BUV737	SJ25C1	612756	BD Biosciences
HLA-DR-PerCP-Cy5.5	L243	339194	BD Biosciences
PD-1-PerCP-Cy5.5	EH12.1	561273	BD Biosciences
cMAF-PE	sym0F1	12-9855-42	Thermo Fisher
IRF8-PE	H31-644	566373	BD Biosciences
pSTAT5-PE-efluor610	SRBCZX	61-9010-42	Thermo Fisher
pSTAT6-PE-eFluro610	CHI2S4N	61-9013-41	Thermo Fisher
HIF1a-AF488	546-16	359707	Biolegend
BATHC2	16B10B53	695602	Biolegend
MEF2c	OTI4F7	MA5-25477	Thermo Fisher
pSTAT2	D3P2P	88410S	Cell Signaling
c-MYC	9E10	NB600302SS	Fisher Scientific
FOSL2	2B2	H00002355-M03	Thermo Fisher
Smad2	D43B4	5339T	Cell Signaling
BCL-6 PE-Cy7	K112-91	563582	BD Biosciences
NFkB	5D10D11	MA5-15870	Thermo Fisher
JunB	512313	MAB4456-SP	R&D Systems

Supplementary Table 4 – Pearson correlation coefficient between inferred TF activity and PD-1 expression in MPeM, MPM and PBMC $CD8^{+}$ T cells.

TF	MPeM $CD8^{+}$ T cells	MPM $CD8^{+}$ T cells	PBMC $CD8^{+}$ T cells
JUN	0.90	-0.31	0.09
FOS	0.90	-0.07	0.07
PBX3	-0.64	-0.08	-0.06
MYC	0.77	0.02	-0.16
RARA	0.78	0.22	-0.13
SMAD2	-0.79	-0.54	-0.21
VDR	0.82	0.37	0.07
SP1	-0.73	0.68	0.22
RFX5	0.69	0.52	0.27
ETS1	0.70	-0.29	-0.32
EPAS1	-0.61	-0.52	-0.31
E2F4	-0.65	0.00	0.25
NFE2L2	-0.91	0.26	0.06
HIF1A	0.80	0.69	0.51
TCF25	0.90	0.52	0.29
CTCF	0.80	0.61	-0.13
MEF2A	-0.63	-0.46	-0.20
MEF2C	-0.82	-0.59	0.17
GATA3	0.67	0.35	-0.42
MYB	0.78	0.37	0.18
E2F1	0.77	-0.17	-0.53
STAT1	0.65	-0.33	0.24
EBF1	-0.21	0.67	0.41
RBPJ	-0.68	-0.22	0.07
IRF4	-0.92	-0.23	-0.04
FOXP1	-0.77	-0.48	-0.14
ESR1	-0.76	-0.20	-0.25
RELA	-0.10	-0.60	-0.35
MITF	0.68	0.13	-0.34
ONECUT1	-0.79	-0.04	0.14
LEF1	0.72	0.54	-0.24
MAFF	0.25	0.63	0.00
MAFG	-0.83	-0.43	-0.18
PBX2	-0.62	0.42	-0.17
MEIS2	0.82	0.14	0.37
THAP11	0.23	0.62	-0.08
SOX6	-0.82	-0.27	-0.01

EGR1	-0.90	-0.03	0.14
SRF	0.72	0.44	-0.20
ATF1	-0.60	-0.66	-0.45
ELK1	-0.68	-0.48	0.34
RELB	-0.67	-0.59	-0.53
FOXO1	-0.66	-0.62	-0.20
IRF1	0.77	0.54	-0.03
CEBPB	0.67	0.00	-0.02
REL	0.74	-0.51	-0.36
ZNF263	-0.80	-0.02	0.26
YY1	0.67	0.53	-0.24
FOXM1	-0.72	-0.28	0.23
TBP	0.69	-0.05	-0.22
NR5A2	0.62	-0.20	0.40
RFX1	0.74	-0.57	-0.53
IRF3	0.80	0.46	-0.22
USF1	0.82	-0.33	0.42
FOSL2	-0.88	-0.53	0.07
SMAD3	-0.60	-0.09	0.08
ESR2	-0.70	0.15	-0.16
CEBPD	-0.84	-0.01	0.22
NFKB2	0.46	0.41	0.61
ZFX	0.77	-0.48	0.22
BCL3	0.89	0.25	-0.09
MYBL2	-0.85	0.41	-0.23
RUNX1	-0.71	-0.49	0.07
NFIC	-0.84	0.60	0.26
SP3	0.80	-0.46	-0.05
AR	0.70	-0.35	-0.20
NFYB	-0.64	0.65	-0.05
SREBF1	0.64	0.09	0.03
E2F6	0.42	0.62	0.07
TP73	-0.82	-0.55	-0.13
CREB3	0.72	0.64	-0.23
FOXP3	-0.87	-0.09	-0.18
BACH2	-0.63	0.02	-0.09
TP63	-0.64	-0.04	-0.29
ATF7	-0.48	-0.65	0.02
STAT3	-0.73	-0.24	0.29
FLI1	-0.69	0.12	0.03

AHR	0.07	-0.61	-0.37
ELK4	-0.68	-0.09	-0.26
STAT6	0.89	-0.71	0.29
FOXL2	-0.83	0.08	0.38
SMARCC2	-0.78	-0.43	0.00
GATA6	0.82	-0.21	-0.13
ATF3	-0.75	0.77	0.01
KLF6	-0.80	0.32	0.23
HINFP	-0.78	-0.29	0.31
HMBOX1	-0.87	-0.33	0.03
LYL1	0.93	0.58	0.10
TCF7	0.87	0.62	0.22
RFXAP	0.23	0.69	-0.10
POU5F1	-0.90	-0.69	0.12
FOXK2	-0.66	-0.48	0.40
RUNX2	-0.77	-0.50	0.06
THAP1	-0.87	0.12	0.29
NR2F6	0.60	0.66	0.05
KLF5	0.62	0.17	-0.17
FOSL1	-0.65	-0.63	-0.37
TCF3	-0.81	-0.08	0.17
NRF1	-0.71	0.53	-0.58
ZBTB33	0.86	0.49	-0.03
IKZF1	0.80	-0.07	0.39
SNAPC4	-0.78	-0.48	-0.58
MEIS1	0.79	0.39	-0.16
STAT5B	0.79	0.63	0.05
MNT	0.07	-0.72	-0.09
ZBED1	0.46	-0.73	0.28
ZHX2	0.75	-0.07	-0.02
TWIST1	0.61	-0.11	0.41
ZKSCAN1	0.95	-0.31	-0.25
REST	-0.79	0.64	0.03
NCOA1	-0.71	-0.32	-0.17
NCOR2	-0.23	-0.35	-0.74
CLOCK	0.79	-0.71	-0.38
ARID1B	-0.82	0.23	0.36
TFAP4	0.62	0.26	0.03
SMAD1	0.72	0.31	-0.34
GABPA	-0.76	-0.17	-0.33

ETV6	-0.70	-0.09	-0.07
TFDP1	0.52	0.61	-0.06
NR2C2	0.67	-0.18	0.15
SP4	0.57	0.65	0.10
E2F5	-0.85	-0.39	-0.26
MAFK	0.80	-0.08	0.27
MAF	0.93	-0.45	0.08
KLF13	0.72	0.23	-0.59
CREB3L1	0.76	0.43	0.63
NCOA2	-0.72	0.40	0.46
KLF1	0.92	0.20	0.46
MTA2	-0.79	0.28	-0.17
SOX13	0.74	-0.52	0.30
FOXJ2	0.78	-0.38	-0.09
ARID3A	-0.80	-0.69	-0.06
ID3	-0.81	-0.24	0.18
ZNF143	-0.78	-0.16	0.30
ZNF217	0.89	-0.07	-0.06
EOMES	-0.66	0.48	-0.11
MIER1	-0.66	0.50	-0.31
SMARCC1	0.38	0.75	0.39
SMAD5	-0.82	0.13	-0.15
TGIF2	-0.92	0.27	0.37

SI References

1. L. Garcia-Alonso, C. H. Holland, M. M. Ibrahim, D. Turei, J. Saez-Rodriguez, Benchmark and integration of resources for the estimation of human transcription factor activities. *Genome Res* **29**, 1363-1375 (2019).
